# Luteolin Alleviates Oxidative Stress in Chronic Obstructive Pulmonary Disease Induced by Cigarette Smoke via Modulation of the TRPV1 and CYP2A13/NRF2 Signaling Pathways

**DOI:** 10.3390/ijms25010369

**Published:** 2023-12-27

**Authors:** Lina Zhou, Tunyu Jian, Yan Wan, Rizhong Huang, Hailing Fang, Yiwei Wang, Chengyuan Liang, Xiaoqin Ding, Jian Chen

**Affiliations:** 1 Jiangsu Key Laboratory for the Research and Utilization of Plant Resources, Institute of Botany, Jiangsu Province and Chinese Academy of Sciences, Nanjing 210014, China; linazhou1124@163.com (L.Z.); jiantunyu1986@163.com (T.J.); liangcy618@cnbg.net (C.L.); 2 School of Pharmacy, Nanjing University of Chinese Medicine, Nanjing 210023, China; wany998061@163.com (Y.W.); 20210615@njucm.edu.cn (R.H.); fanghailing2013@163.com (H.F.); yiweiwang@njucm.edu.cn (Y.W.)

**Keywords:** cigarette smoke, chronic obstructive pulmonary disease (COPD), alveolar epithelial (A549) cells, luteolin, TPRV1, CYP2A13

## Abstract

The current study aims to investigate the therapeutic potential of luteolin (Lut), a naturally occurring flavonoid found in various medicinal plants, for treating chronic obstructive pulmonary disease (COPD) through both in vitro and in vivo studies. The results demonstrated that Lut increased body weight, reduced lung tissue swelling and lung damage indices, mitigated systemic oxidative stress levels, and decreased alveolar fusion in cigarette smoke (CS)- and lipopolysaccharide (LPS)-induced COPD mice. Additionally, Lut was observed to downregulate the expression of the TRPV1 and CYP2A13 proteins while upregulating SIRT6 and NRF2 protein expression in CS + LPS-induced COPD mice and cigarette smoke extract (CSE)-treated A549 cells. The concentrations of total reactive oxygen species (ROS) and mitochondrial ROS in A549 cells induced by CSE significantly increased. Moreover, CSE caused a notable elevation of intracellular Ca^2+^ levels in A549 cells. Importantly, Lut exhibited inhibitory effects on the inward flow of Ca^2+^ and attenuated the overproduction of mitochondrial and intracellular ROS in A549 cells treated with CSE. In conclusion, Lut demonstrated a protective role in alleviating oxidative stress and inflammation in CS + LPS-induced COPD mice and CSE-treated A549 cells by regulating TRPV1/SIRT6 and CYP2A13/NRF2 signaling pathways.

## 1. Introduction

Chronic obstructive pulmonary disease (COPD), a persistent respiratory lung ailment, significantly impairs the quality of life due to its severe systemic symptoms and associated conditions [1]. According to the WHO report, COPD currently affects nearly 400 million people, posing a substantial threat to public health, and is anticipated to become the third leading cause of global mortality by 2030 [2]. This is largely due to challenges in the early identification of COPD airflow limitations [3]. Growing evidence indicates that an elevated level of oxidative stress in the lungs is crucial in driving COPD through a complex network of mechanisms [4,5]. While infection, abnormal lung development, and genetic factors can contribute to COPD, cigarette smoke (CS) remains the predominant risk factor for its onset and progression. CS significantly promotes chronic airway inflammation, excessive mucus production, lung tissue damage, and the remodeling of small airways [5,6,7,8]. These processes may exacerbate systemic comorbidities or impose irreversible airway constraints [9,10]. The gas mixture from CS contains over 4500 hazardous substances, including carbon monoxide, nicotine, oxidants, and tiny particulate matter [11]. In COPD patients, the high concentration of oxidants inhaled from CS can disrupt the delicate balance between oxidants and antioxidants, leading to oxidative stress, particularly in the lungs [12,13,14,15]. While the survival rate of COPD patients has increased in recent decades, and studies show promising avenues for potential antibody-based treatments in managing COPD and ultimately improving patient outcomes, there still remains an urgent need to discover novel therapies in the treatment of COPD [16,17].

The transient receptor potential vanilloid 1 (TRPV1) protein is expressed in airway epithelial cells and can be activated by capsaicin, oxidative stress, or external environmental stimuli such as cigarette smoke [18,19,20]. Numerous studies have suggested that TRPV1 plays a role in pathological lung processes, including chronic inflammation, fibrosis, and edema [21]. Moreover, it is asserted that TRPV1 contributes to the initiation of lung damage induced by reactive oxygen species (ROS) [22,23,24].

In addition to TRPV1, the respiratory system contains the enzyme Cytochrome P450 Family 2 Subfamily A Member 13 (CYP2A13), responsible for metabolizing substances from the environment, including those present in cigarette smoke, particularly nicotine [25,26]. Upon exposure to CS, CYP2A13 in the lungs is compelled to overexpress, generating reactive oxygen species (ROS) and active metabolites that harm the respiratory system [27,28]. Despite these findings, the exact mechanism through which TRPV1 and CYP2A13 contribute to the progression of oxidative stress initiated by CS in COPD remains uncertain.

Natural flavonoids like luteolin (Lut) are found in medicinal plants, vegetables, and fruits [29]. Honeysuckle is a vibrant source of Lut [29,30,31]. Lut demonstrates various physiological functions, including anti-oxidative and anti-inflammatory properties [32,33,34]. Researchers have observed that Lut can inhibit injury and apoptosis in normal human bronchial epithelial cells induced by cigarette smoke extract (CSE) [35]. Given its involvement in oxidation-reduction reactions, Lut has shown therapeutic effects in various lung diseases [36,37]. Considering the significant roles of TRPV1 and CYP2A13 in oxidative stress [21,27,28], we hypothesize that Lut may alleviate CS-induced oxidative stress in COPD mice by modulating the TRPV1 and CYP2A13 pathways. While previous studies have demonstrated the therapeutic effects of Lut in COPD [37,38], our work aims to explore the relationship between TRPV1, CYP2A13, and other essential effectors following Lut administration. This is performed to validate and comprehend another potential mechanism of Lut in CS-induced COPD.

In this study, we conducted comprehensive research on the potential mechanism of luteolin’s (Lut) regulation of TRPV1 and CYP2A13 in the treatment of COPD, using cigarette smoke extract (CSE)-treated A549 cells and cigarette smoke (CS)- and lipopolysaccharide (LPS)-induced COPD mouse models. Our findings propose an alternative mechanism of action for Lut, intending to restore redox balance and counteract the impact of CS on TRPV1 and CYP2A13. This approach holds promise for addressing various clinical symptoms associated with COPD. This study unravels a distinctive pathway through which Lut intervenes in COPD pathogenesis, offering a novel perspective on combating this complex ailment.

## 2. Results

### 2.1. Lut Relieved CSE-Induced Oxidative Stress of A549 Cells

**Figure 1:**
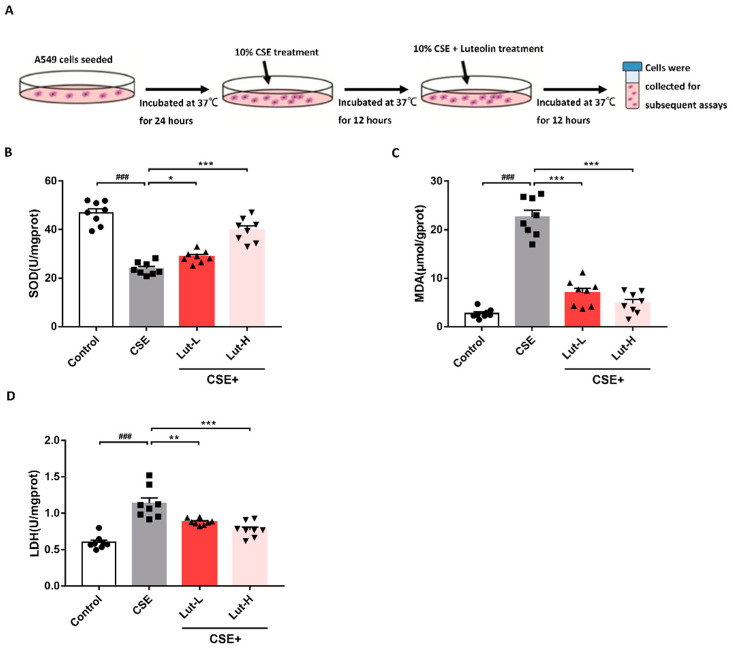
Lut relieved CSE-induced oxidative stress of A549 cells. The schematic of cell experimental protocol (**A**). The levels of SOD (**B**), MDA (**C**), and LDH (**D**) in the cells were measured using commercial kits. Data were presented as mean ± SEM, *n* = 8 per group. ^###^ *p* < 0.001 vs. cells in the Control group. * *p* < 0.05, ** *p* < 0.01, *** *p* < 0.001 vs. cells in the CSE group.

To investigate the impact of Luteolin on oxidative stress induced by CSE exposure, oxidative stress-related indicators were measured after treatment with 15 and 30 μM of Lut for 12 h. Elevated levels of lactate dehydrogenase (LDH) and malondialdehyde (MDA) were observed in the CSE group compared to the Control group. However, the generation of MDA and LDH was suppressed by Lut treatment (Figure 1C,D), indicating that Lut significantly attenuated the generation of MDA and LDH induced by CSE (*p* < 0.01, *p* < 0.001). Subsequently, superoxide dismutase (SOD) activity among the four groups was measured using fluorescence spectrophotometry. The results showed a notable decrease in SOD activity in the CSE group compared to the Control group (*p* < 0.001). In contrast, the CSE + Lut groups exhibited a significant increase in SOD activity (*p* < 0.05, *p* < 0.001, Figure 1B). Therefore, Lut inhibited CSE-induced oxidative stress in A549 cells.

### 2.2. Lut Lessened ROS Generation in A549 Cells Induced by CSE

**Figure 2:**
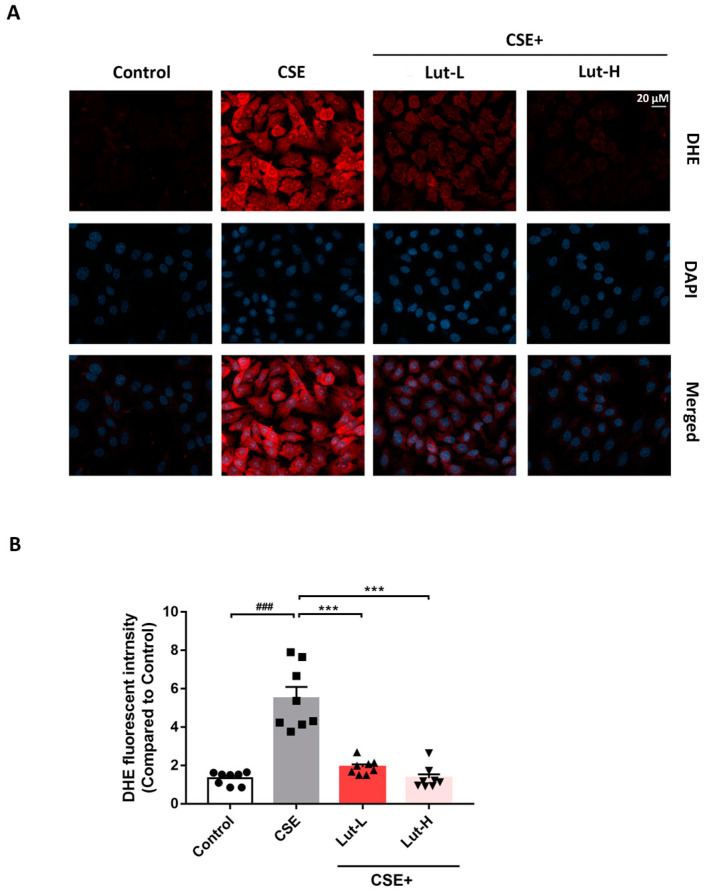
Lut lessened ROS generation in A549 cells induced by CSE. ROS in A549 cells incubated with or without 10% CSE or Lut (15, 30 μM) for 24 h was detected by DCFH-DA labeling (bar = 20 μm) (**A**). The quantification chart is on the right side (**B**). Data were presented as mean ± SEM. ^###^ *p* < 0.001 vs. cells in the Control group. *** *p* < 0.001 vs. cells in the CSE group.

**Figure 3:**
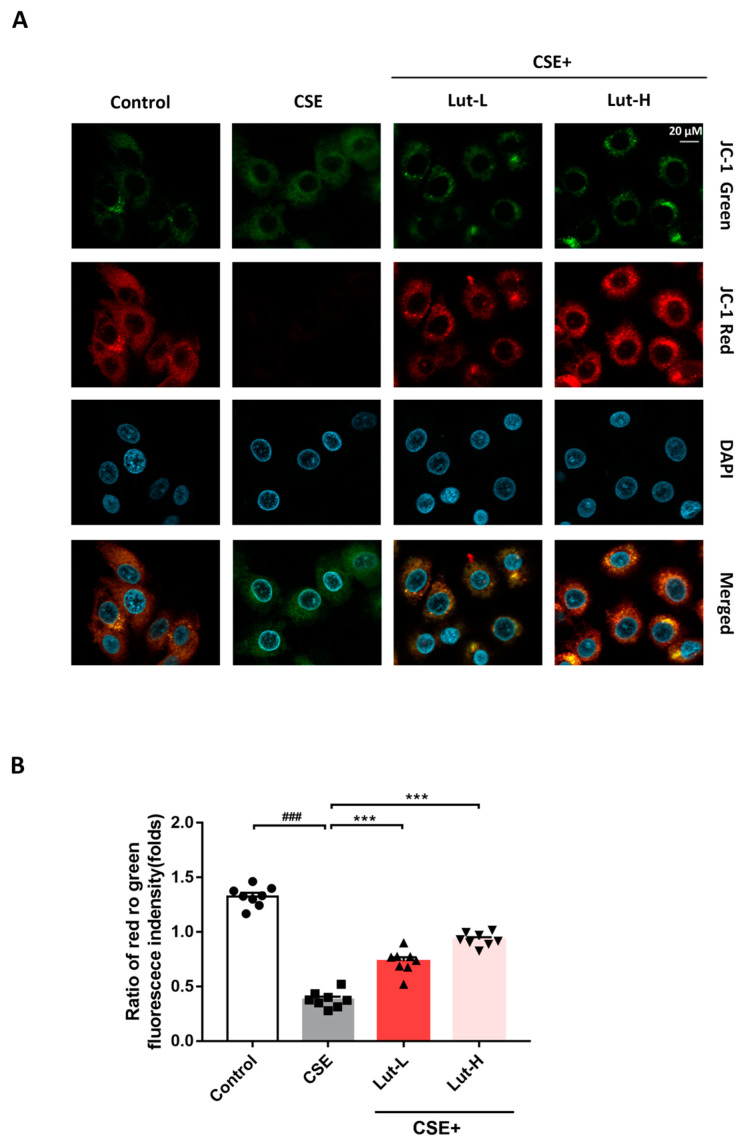
Lut lessened mitochondrial ROS activity in A549 cells induced by CSE. ROS in A549 cells incubated with or without 10% CSE or Lut (15, 30 μM) for 24 h detected by Mito SOX Red labeling (bar = 20 μm) (**A**). The quantification chart is on the right side (**B**). Data were presented as mean ± SEM. ^###^ *p* < 0.001 vs. cells in the Control group. *** *p* < 0.001 vs. cells in the CSE group.

To investigate intracellular ROS, the fluorescent probe 2′,7′-dichlorodihydrofluorescein diacetate (DCFH-DA), known for detecting ROS, was utilized (Figure 2). The CSE group exhibited enhanced red fluorescence compared with the Control group, indicating that CSE exposure induced ROS. Additionally, when cells were treated with different concentrations of Luteolin, images displayed a weaker red fluorescence signal, suggesting that Lut significantly reversed the accumulation of superoxide anions in cells induced by CSE treatment, thereby alleviating oxidative stress reactions (Figure 2). Furthermore, intracellular mitochondrial ROS (Mito-Sox) were observed to be significantly elevated in CSE-treated A549 cells (*p* < 0.01). These effects were effectively inhibited by Lut treatment (*p* < 0.01). These results demonstrate that CSE significantly increased the level of oxidative stress in A549 cells, and Lut could effectively reverse this trend (Figure 3).

### 2.3. Lut Attenuated CSE-Induced Ca^2+^ Influx

**Figure 4:**
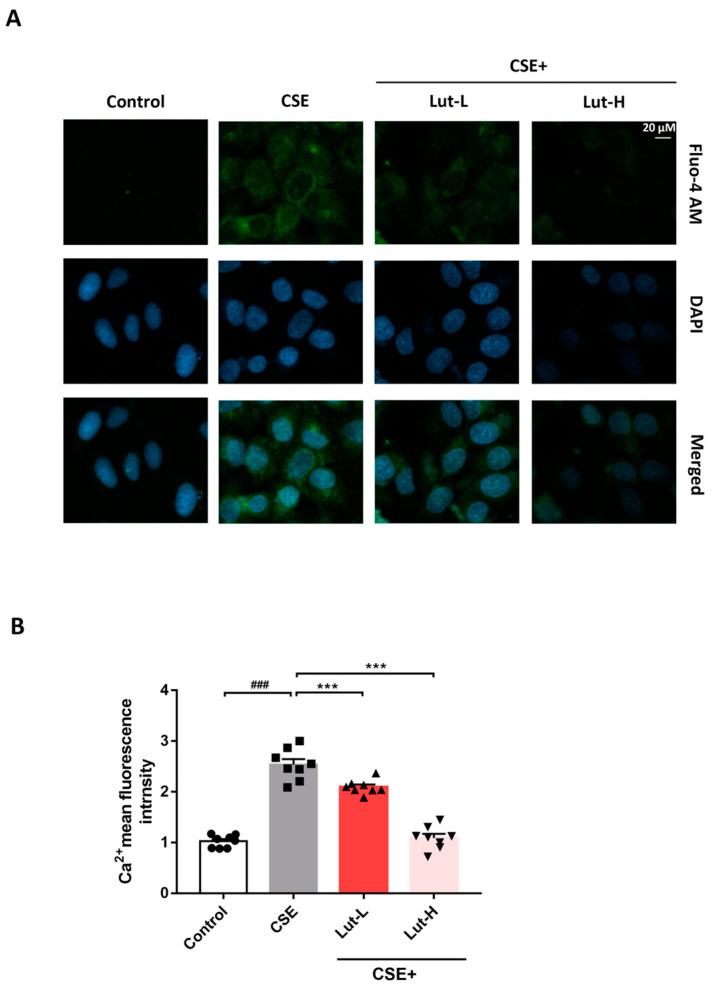
Lut attenuated CSE-induced Ca^2+^ influx. Ca^2+^ in A549 cells incubated with or without 10% CSE or Lut (15, 30 μM) for 24 h was detected by Fluo-4 AM labeling (bar = 20 μm) (**A**). The quantification chart is on the right side (**B**). Data were presented as mean ± SEM. ^###^ *p* < 0.001 vs. cells in the Control group. *** *p* < 0.001 vs. cells in the CSE group.

Substantial evidence indicates that the flux of calcium ions (Ca^2+^) and the generation of mitochondrial ROS are correlated under certain conditions, suggesting a mutual influence between cellular redox status and calcium homeostasis [39,40]. To further elucidate the interaction between Ca^2+^ homeostasis and mitochondrial ROS generation, we investigated whether there was a Ca^2+^ homeostasis imbalance in 549 cells exposed to CSE. Following exposure to CSE, the intracellular Ca^2+^ levels were elevated compared to those in the Control group (*p* < 0.001), consistent with heightened oxidative stress in CSE-treated cells. In contrast, both high and low doses of Lut treatment reversed the CSE-induced increase in Ca^2+^ levels (*p* < 0.001). Taken together, these results indicate that oxidative stress could increase Ca^2+^ influx in A549 cells, while Lut treatment relieved the influx of Ca^2+^ (Figure 4).

### 2.4. Lut Regulated TRPV1/Sirtuin 6 (SIRT6) and CYP2A13/Nuclear Factor E2-Related Factor 2 (NRF2) to Mitigate Oxidative Stress in CSE-Exposed A549 Cells

**Figure 5:**
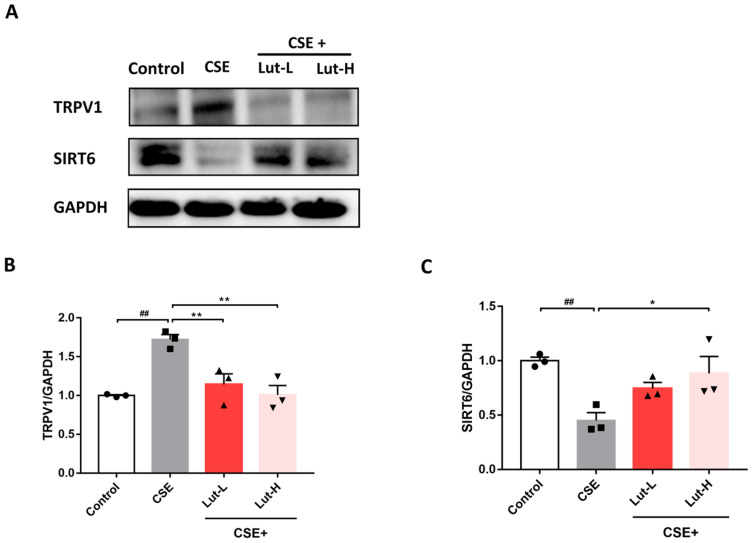
Lut regulated TRPV1/SIRT6 to alleviate oxidative stress in CSE-exposed A549 cells. Representative bands (**A**) and protein levels of TRPV1 (**B**) and SIRT6 (**C**) in A549 cells incubated with or without 10% CSE or Lut (15, 30 μM) for 24 h (*n* = 3). Data were presented as mean ± SEM. ^##^ *p* < 0.01 vs. cells in the Control group. * *p* < 0.05, ** *p* < 0.01 vs. cells in the CSE group.

Ca^2+^ plays a crucial role as a second messenger in various cellular signaling pathways [41,42]. TRPV1, as a calcium permeation channel, is a pathway for Ca^2+^ entry. Moreover, the TRPV1/SIRT6 pathway is vital to defense mechanisms against oxidative stress. Both TRPV1 inhibitors and gene deletion have demonstrated evidence of reducing oxidative stress and increasing the expression of other antioxidant genes [43]. Therefore, we further investigated the expression of TRPV1 and SIRT6 in A549 cells. In this study, cells treated with CSE exhibited notably high expression levels of TRPV1 (*p* < 0.01), while the expression of SIRT6 (*p* < 0.01) decreased compared to the Control group. Following Lut treatment, a significant reduction in TRPV1 expression (*p* < 0.01) was observed, along with an increase in the expression of SIRT6 (*p* < 0.05, Figure 5).

**Figure 6:**
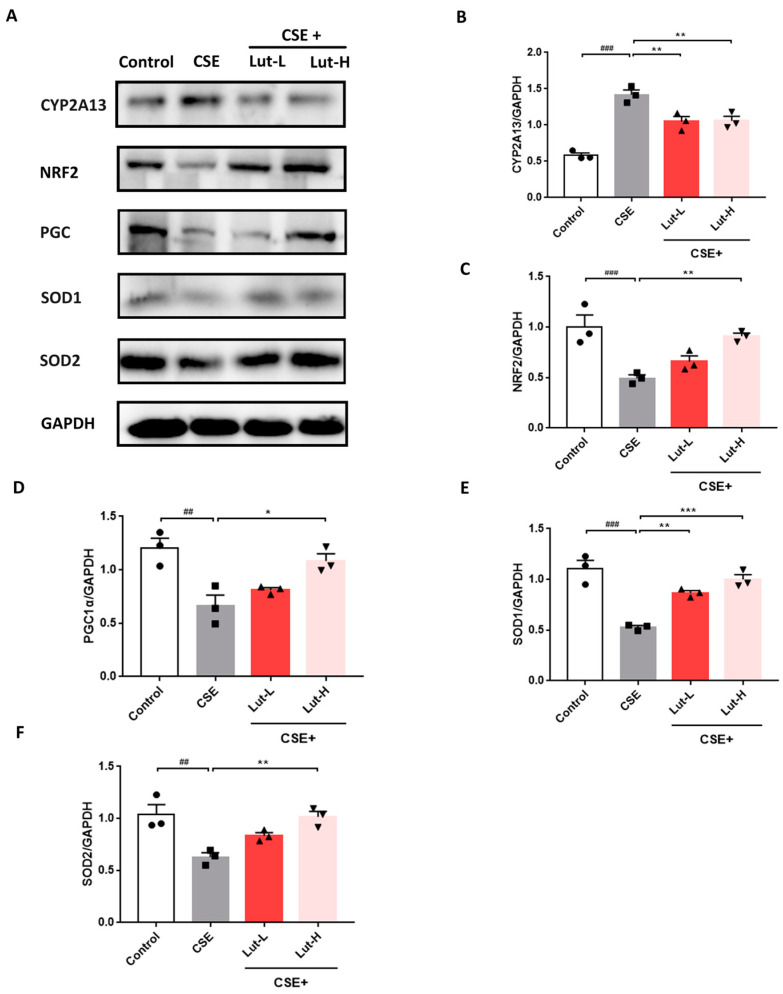
Lut regulated CYP2A13/NRF2 to alleviate oxidative stress in CSE-exposed A549 cells. Representative bands (**A**) and protein levels of CYP2A13 (**B**), NRF2 (**C**), PGC1α (**D**), SOD1 (**E**), and SOD2 (**F**) in A549 cells incubated with or without 10% CSE or Lut (15, 30 μM) for 24 h (*n* = 3). Data were presented as mean ± SEM. ^##^ *p* < 0.01, ^###^ *p* < 0.001 vs. cells in the Control group. *p < 0.05, ** *p* < 0.01, *** *p* < 0.001 vs. cells in the CSE group.

It is generally assumed that CYP2A13 is primarily present in the human respiratory system, mediating the metabolism of cigarette smoke and the generation of toxic metabolites, such as ROS [44]. Additionally, we examined the amount of CYP2A13 protein in cells to determine if Lut may decrease tobacco metabolism in A549 cells. Compared to the Control group, Figure 6 shows that the CSE group displayed elevated expression levels of CYP2A13 (*p* < 0.001). However, after Lut administration, there was a significant reduction in the level of CYP2A13 (*p* < 0.01). NRF2 is a transcription factor that maintains oxidative/antioxidant balance [45]. Concurrently with the increased expression of CYP2A13, exposure to CSE resulted in the downregulation of NRF2 expression (*p* < 0.001), while both high and low doses of Lut treatment resulted in the upregulation of NRF2 protein (*p* < 0.01). The expression levels of peroxisome proliferator-activated receptor gamma coactivator-1α (PGC1α) (*p* < 0.01), SOD1 (*p* < 0.001), and SOD2 (*p* < 0.01) were notably reduced in cells of the CSE group compared to those in the Control group. However, the administration of Lut significantly reversed these trends (Figure 6; *p* < 0.01, *p* < 0.001). These findings indicate a significant antioxidant effect of Lut in COPD.

### 2.5. Lut Mitigated the Weight Loss and Lung Damage Caused by Cigarette Smoke in COPD Mice

**Figure 7:**
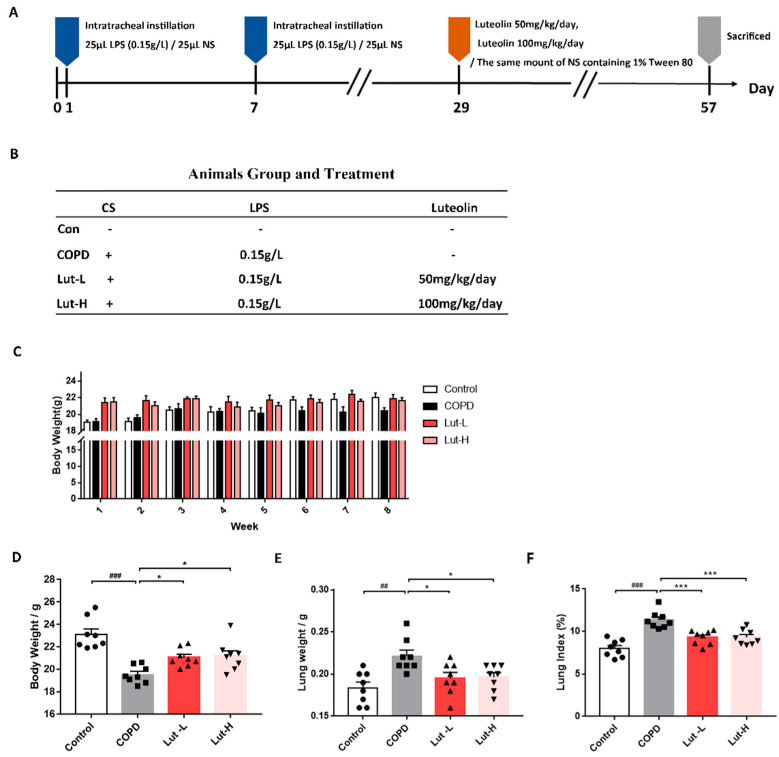
The diagram of the animal experiment process (**A**), group, and treatment (**B**). Effects of Lut on body weight (**C**), lung weight (**D**), and lung index (**E**) in normal mice (Control) or CS + LPS-exposed mice treated with 50 and 100 mg/kg Lut (*n* = 8). Data were presented as mean ± SEM. ^##^ *p* < 0.01, ^###^ *p* < 0.001 vs. cells in the Control group. * *p* < 0.05, *** *p* < 0.001 vs. cells in the COPD group.

In the in vivo study, COPD mice induced by CS + LPS exposure were employed to investigate the therapeutic effect of Lut. The specific dosing procedure is illustrated in Figure 7A. In comparison to the Control group, mice exposed to CS + LPS exhibited significant decreases in body weight along with an increase in lung tissue weight and lung index, indicating signs of pulmonary edema (*p* < 0.01, *p* < 0.001). Lut treatment demonstrated the ability to reverse weight loss in mice from the COPD group while significantly reducing lung weight (*p* < 0.05, *p* < 0.001).

**Figure 8:**
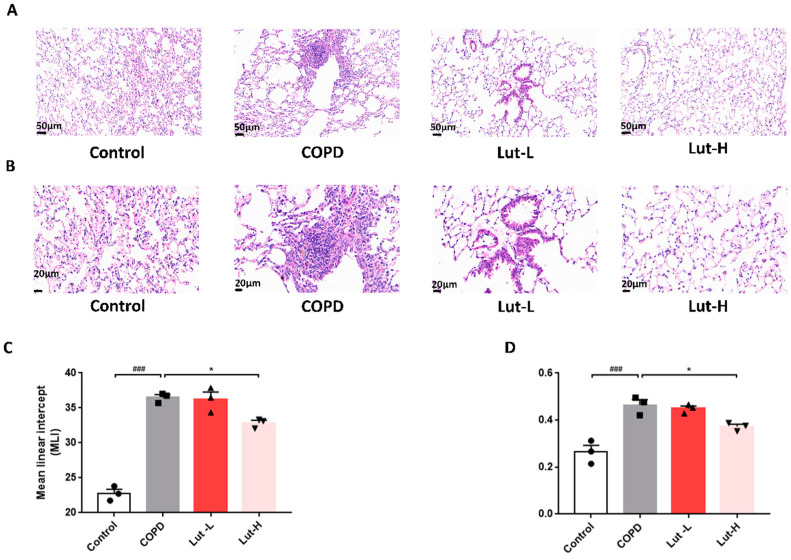
Effects of Lut treatment on CS + LPS-induced lung emphysema. Representative histopathological images (bar = 50 μm, (**A**); bar = 20 μm, (**B**)). The mean MLI is defined as the damage of alveolar walls (**C**), and the DI is defined as airspace enlargement (**D**) (*n* = 3). Data were presented as mean ± SEM. ^###^ *p* < 0.001 vs. the Control group. * *p* < 0.05 vs. the COPD group.

Figure 8 illustrates the histological changes in the lungs after exposure to CS + LPS and subsequent Lut therapy. Histological analysis showed that mice exposed to fresh air exhibited fully developed and uniform alveolar structures with rare inflammatory cell infiltration. The COPD mice exhibited a marked reduction in alveolar count within their lungs and increased pulmonary bulla formation. Prominent pathological changes were evident, including the fusion of alveoli and narrowing of alveolar spaces (Figure 8A,B). Nevertheless, treatment with Lut effectively reversed these pathological changes in a manner dependent on the dosage (Figure 8A,B). Essential parameters for assessing lung injury include mean linear intercept (MLI) values and destructive index (DI) values. The MLI and DI values in the CS + LPS-exposed mice were higher than those in Control mice. After treatment with Lut, the heightened values in COPD mice exhibited a notable decrease, as illustrated in Figure 8C,D (*p* < 0.05).

### 2.6. Lut Attenuated CS + LPS-Induced Systemic Oxidative Stress in Mice

**Figure 9:**
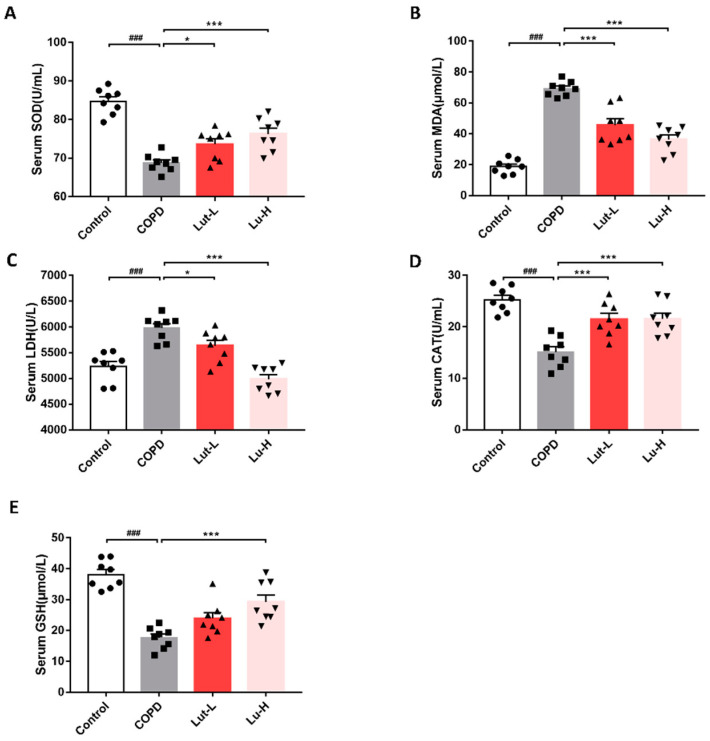
Lut attenuates CS + LPS-induced systemic oxidative stress in mice. C57BL/6J mice were randomly divided into four groups: in the Control group, mice were exposed to air and received 0.9% NaCl solution containing 1% Tween 80. In the COPD group, mice were exposed to CS + LPS and received 0.9% NaCl solution containing 1% Tween 80. In the two COPD + Lut groups, mice were exposed to CS + LPS and received 50 mg/kg/day or 100 mg/kg/day of Lut once daily by oral gavage. The levels of serum SOD (**A**), MDA (**B**), LDH (**C**), CAT (**D**), and GSH (**E**) in mice using commercial kits (*n* = 8). Data were presented as mean ± SEM, *n* = 8 per group. ^###^ *p* < 0.001 vs. the Control group. * *p* < 0.05, *** *p* < 0.001 vs. the COPD group.

As illustrated in Figure 9, the total serum levels of SOD, catalase (CAT), and glutathione (GSH) were found to decrease in the COPD mice, while levels of MDA and LDH were significantly higher compared to the Control mice (*p* < 0.001). However, upon the administration of Lut, a significant reduction was observed in MDA and LDH levels (*p* < 0.05, *p* < 0.001), accompanied by an increase in SOD, CAT, and GSH levels. (*p* < 0.05, *p* < 0.001). This implies that Lut could assist mice in mitigating systemic oxidative damage triggered by CS + LPS.

### 2.7. Lut Treatment Modulated TRPV1/SIRT6 and CYP2A13/NRF2 Pathways to Reduce Oxidative Stress in CS + LPS-Exposed COPD Mice

**Figure 10:**
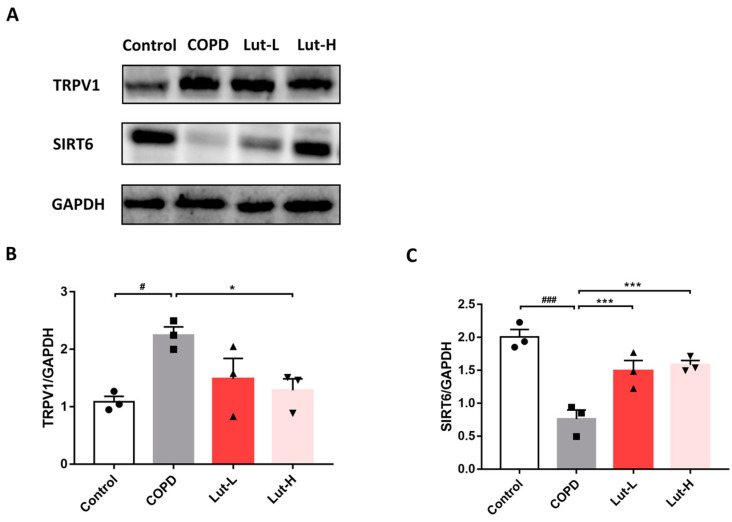
Lut regulated TRPV1/SIRT6 to alleviate oxidative stress in CS + LPS-exposed COPD mice. Representative bands (**A**) and protein levels of TRPV1 (**B**) and SIRT6 (**C**) in mouse lung tissue (*n* = 3). Data were presented as mean ± SEM. ^#^ *p* < 0.05, ^###^ *p* < 0.001 vs. the Control group. * *p* < 0.05, *** *p* < 0.001 vs. the COPD group.

According to Figure 10, mice after CS + LPS exposure showed a significant increase in TRPV1 expression (*p* < 0.05) and a significant decrease in SIRT6 expression (*p* < 0.001). In contrast, when compared to the COPD group, Lut notably enhanced SIRT6 expression and markedly reduced TRPV1 expression (*p* < 0.05, *p* < 0.001).

**Figure 11:**
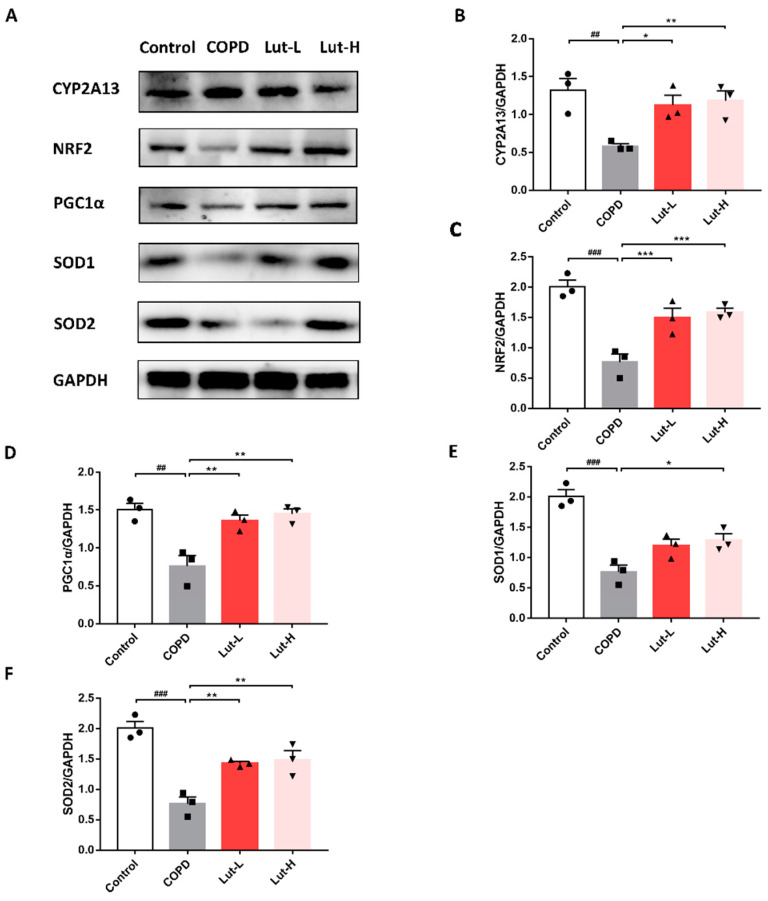
Lut regulated CYP2A13/NRF2 to alleviate oxidative stress in CS + LPS-exposed COPD mice. Representative bands (**A**) and protein levels of CYP2A13 (**B**), NRF2 (**C**), PGC1α (**D**), SOD1 (**E**), and SOD2 (**F**) in mouse lung tissue (*n* = 3). Data were presented as mean ± SEM. ^##^ *p* < 0.01, ^###^ *p* < 0.001 vs. the Control group. * *p* < 0.05, ** *p* < 0.01, *** *p* < 0.001 vs. the COPD group.

As seen in Figure 11, animals in the CS + LPS-exposed COPD group displayed downregulation of NRF2, PGC1α, SOD1, and SOD2 (*p* < 0.01, *p* < 0.001), and CYP2A13 levels were increased in the COPD group compared to the Control group (*p* < 0.01). Nevertheless, Lut was able to reduce the expression of CYP2A13 (*p* < 0.05, *p* < 0.01), while notably increasing the levels of proteins that had decreased in COPD mice (*p* < 0.05, *p* < 0.01, *p* < 0.001).

## 3. Discussion

Flavonoids derived from natural sources are acknowledged for their outstanding anti-inflammatory and antioxidant characteristics and are often utilized in COPD treatment [46]. Quercetin, a natural flavonoid derived from various plants, has been the subject of comprehensive research exploring its effects and mechanisms in COPD management [47]. Luteolin, similar to Quercetin in its natural flavonoid composition but differing by only one hydroxyl group, has been highlighted in previous studies for its therapeutic potential in treating COPD. In our present study, we also observed the anti-COPD effects of Luteolin, which are consistent with findings from prior research [37,38]. The mentioned studies have revealed that Lut treatment for COPD might involve modifying the epidermal growth factor receptor (EGFR), matrix metalloproteinase (MMP), and NADPH oxidase (NOX4). However, our specific objective was to establish correlations between TRPV1, CYP2A13, and other crucial factors subsequent to Lut administration in COPD. This exploration unveils a unique pathway through which Lut intervenes in the pathogenesis of COPD, presenting a new perspective on managing this intricate condition. This study aimed to validate and enhance our understanding of an additional potential mechanism of Lut in COPD induced by CS exposure.

Considerable evidence in the previous literature supports that cigarette smoke is a primary source of ROS [48,49]. An excess of ROS leads to oxidative stress that can trigger the development of pathological changes in the lungs of COPD patients [50,51]. Unfortunately, the precise pathogenesis of the disease remains unclear, limiting the development of effective therapeutic drugs for COPD. According to previous findings and clinical results about COPD therapy [43,52], we expected that Lut could inhibit the oxidative stress response induced by CS and be helpful as a therapeutic agent to treat COPD. The present study employed CSE to establish an in vitro model using A549 cells and CS + LPS exposure to develop an in vivo model using C57BL/6J mice, and potential antioxidant effects were observed in both models.

Similarly, our results demonstrated that the CSE group cells exhibit a higher production of ROS, as evidenced by enhanced red fluorescence signals. Moreover, intracellular Mito-Sox was increased in A549 cells cocultured with CSE. This is consistent with the finding that cigarette smoke is responsible for the increase in ROS. Antioxidant enzymes such as SOD are known to be responsible for quenching these ROS and preserving redox homeostasis [53]. Our results demonstrated that cigarette smoke significantly decreased the serum levels of molecules, with a protective effect against excessive oxidations. It is well documented that hydroxyl radicals, released from cigarette smoke, can react with unsaturated fatty acids located on cellular membranes. Subsequently, secondary products are generated by this reaction, resulting in deleterious effects of lipid peroxidation, such as MDA [53,54,55]. CS exposure could deplete antioxidants such as glutathione and activate proteinases, induce oxidative stress, and ultimately lead to lung injury. Oxidative stress can also destroy lipids, nucleic acids, and proteins, further exacerbating oxidative stress [56]. In the COPD model group, the MDA level was higher in the serum of mice and A549 cells than in the control group. These observations indicated that smoking-related oxidative stress further leads to increased peroxidation. Moreover, ROS and lipid peroxidation products can exacerbate COPD by affecting signal transduction mechanisms.

TRPV1 is a chemo-sensor and an essential component of signal transduction cascades involved in pulmonary diseases, including COPD [57,58]. The data provided in this study strongly support the significant involvement of TRPV1 channels in the elevation of oxidative stress after CS exposure. CSE increased intracellular TRPV1 protein content and higher levels of oxidative stress than the Control group, as demonstrated in cell culture experiments. Notably, the mouse model showed overexpression of the TRPV1 protein and significantly higher levels of oxidative stress factors after CS + LPS exposure compared to the Control group, suggesting that this pathway may be necessary within the CS + LPS-induced oxidative stress facilitating COPD.

The activation of the TRPV1 channel can result in an inward flow of Ca^2+^, leading to mitochondrial Ca^2+^ uptake and overload. This fact could be primarily responsible for the shift in ROS messenger function from normality to cellular damage and death signaling [59,60,61]. After CSE exposure, Lut treatment significantly reversed the substantial cell damage caused by the pronounced intracellular Ca^2+^ influx in A549 cells. These findings shed light on the role of the TRPV1 channel in the progression of CS-induced COPD. Our results also showed that Lut attenuated CS + LPS-induced lung swelling and lung injury index in mice. In addition to this, Lut restored CSE-induced SOD enzyme activity and reduced ROS, MDA, and LDH levels in A549 cells. Importantly, expression of TRPV1 was alleviated both in the lung tissue of CS + LPS-exposure-induced COPD mice and in CSE-treated A549 cells after Lut treatment.

SIRT6 belongs to the Sirtuins family of deacetylases that are activated by nicotinamide adenine dinucleotide (NAD^+^), and they are involved in various physiological activities such as oxidative stress. SIRT6 has been shown to reduce emphysema by inhibiting matrix metalloproteinases and regulating the expression of several antioxidant genes [62,63]. Although SIRT6 has been implicated in the etiology of COPD, the underlying processes have yet to be fully elucidated [64]. Our findings demonstrated that reduced SIRT6 was associated with COPD development during CS exposure. Our data reveal that Lut might attenuate cigarette smoke-induced oxidative damage by regulating the TRPV1/SIRT6 pathway in vivo and in vitro.

The metabolic enzyme CYP2A13 is mainly found in the human respiratory system, specifically in the trachea and bronchial epithelial cells [25]. CYP2A13 is crucial for the development of cigarette-related lung swelling and pathological injury [65,66]. Specifically, CYP2A13 metabolizes the nicotine in cigarette smoke to cotinine and reactive metabolites such as nitrogen-derived nitrosamine ketone (NNK) [67,68]. Multiple studies have shown that NNK and/or its metabolites probably can generate hydroxyl and other reactive oxygen species that derive from oxidative stress in lung tissues [27,28,69]. The elevated expression of CYP2A13 might be associated with abnormal cellular activation and increased oxidative stress stimulated by cigarette smoke metabolites. Likewise, we observed abnormal CYP2A13 expression in the lungs of COPD mice and A549 cells treated with CSE, and Lut treatment reversed the abnormal changes.

NRF2 is a major regulator of oxidative stress, responsible for regulating the expression of various antioxidant genes, including SOD and catalase [70,71]. Activation of NRF2 has also been shown to increase the production of factors protecting against oxidative stress in animal models of COPD [72,73]. The disruption of NRF2 leads to emphysema in cigarette smoke-exposed mice [74,75]. Therefore, impaired NRF2 activity is a major risk factor for developing COPD pathogenesis. Moreover, NRF2 can directly control the expression of PGC1α, a master regulator of mitochondrial function and biogenesis [76]. We observed that the expression of downstream antioxidants like SOD1, SOD2, and PGC1α was elevated due to Lut’s inhibition of NRF2, ameliorating lung injury both in vivo and in vitro. Our findings showed that Lut partially corrected the loss in NRF2 protein levels and increased ROS generation caused by CSE in A549 cells.

Previous studies have investigated the potential therapeutic effects of Lut on lung diseases [37,38]. Yapeng Hou et al. highlighted the protective effect of Lut via modulating the cGMP/PI3K pathway in the LPS-induced lung injury model in mice [77]. Here, we used a CS + LPS-induced COPD mouse model and a cellular model exposed to CSE to explore the antioxidant effects of Lut, attempting to connect the crosstalk between the TRPV1/SIRT6 and CYP2A13/NRF2 systems in cigarette smoke-induced COPD. Our findings aim to elucidate the signaling pathways involved in the action of Luteolin regarding COPD. We confirmed that Lut is a bioactive compound with the potential to improve CS-induced COPD. Given the complex etiology of COPD, Lut might be useful as a part of a COPD therapy approach.

## 4. Material and Methods

### 4.1. Reagents

The tar and carbon monoxide concentrations in a complete Hongmei cigarette from Hongta Tobacco Group Limited (Yuxi, China) are 10 mg and 12 mg, respectively. Luteolin, obtained from Solarbio Co., Ltd. (≥98%, Beijing, China), was dissolved in dimethyl sulfoxide (DMSO) for in vitro experiments and 1% Tween 80 for in vivo studies. Kits for measuring MDA and BCA protein assay were sourced from Beyotime Biotechnology (Shanghai, China). Commercial kits for assessing SOD, LDH, CAT, and GSH were procured from Nanjing Jiancheng Bioengineering Institute (Nanjing, China).

The following primary antibodies were used for the Western blotting (WB) assay: Anti-TRPV1 and anti-GAPDH were purchased from Proteintech (Chicago, IL, USA), and anti-CYP2A13 was purchased from Affinity Biosciences (Cincinnati, OH, USA). Anti-SIRT6, anti-SOD1 anti-SOD2, anti-PGC1α), and anti-NRF2 were purchased from Cell Signaling Technology (Danvers, MA, USA). Enhanced chemiluminescence (ECL) detection solution was obtained from Tanon Science and Technology Co., Ltd. (Shanghai, China).

### 4.2. Cigarette Smoke Extract Preparation

On the day of each trial, CSE was freshly generated. In brief, two unfiltered Hongmei cigarettes were burned, and their smoke was drawn into a syringe at a constant rate of 8 mL/s. Subsequently, 10 mL of serum-free medium was forcefully injected into a tube. The pH of the CSE solution was adjusted to 7.4 after sterilization with a 0.22 μm Millipore filter (Bedford, MA, USA). Theoretically, the CSE solution, consisting entirely of 100% CSE, was then diluted to the required concentrations in serum-free media and applied to the cells at various intervals. Figure 1 illustrates the cell experimentation technique.

### 4.3. Animal Model and the Treatment

Male SPF C57BL/6J mice were housed in a temperature-controlled environment (25 ± 2 °C) with a relative humidity of 40–70%. All animals had unrestricted access to standard commercial feed and water. All experimental protocols were conducted under the IACUC guidelines, and all animal trial techniques employed in this study were approved by the Nanjing University of Chinese Medicine’s Animal Care and Use Committee (No. A210403, Nanjing, China). Three groups were established using a random number generator from a pool of 40 male mice: the COPD model group (COPD), the COPD + Lut 50 mg/kg group (Lut-L), and the COPD + Lut 100 mg/kg group (Lut-H). The chosen doses of Lut were based on relevant research [78].

CS + LPS-induced COPD mice were established by intratracheal instillation of LPS combined with CS exposure. Briefly, intratracheal injections of LPS (L2630, Sigma-Aldrich, St Louis, MO, USA, 1 mg in 50 mL saline) were conducted on the first and fourteenth days of the trial. Fresh CS was generated from 20 cigarettes, and the smoke was delivered to a whole-body smoking exposure chamber where mice were passively exposed for 15 min, twice daily, six days per week, for eight weeks. Starting from the 15th week of CS exposure, animals were orally administered excipients (saline containing 1% Tween 80) and varying doses of Lut one hour before CS exposure. 

### 4.4. Blood and Tissue Collection

Body weights were recorded, blood was drawn and centrifuged at 2500× *g* and 4 °C for 15 min to extract serum, and serum was then frozen at −80 °C. Lungs were also collected and weighed. The remaining tissues were immediately frozen in liquid nitrogen and stored at −80 °C for subsequent protein analysis. Additionally, the upper lobe of the right lung’s tissue was promptly fixed in 10% neutral buffered formalin for 24 h.

### 4.5. Antioxidant Detection

To assess the levels of oxidative stress markers, including SOD, MDA, GSH, LDH, and CAT concentrations, appropriate kits were used following the manufacturer’s recommendations. Serum samples were tested, and the plates were read using an automatic plate reader (Molecular Device, Sunnyvale, CA, USA) at the appropriate wavelength.

### 4.6. Histopathological Analysis

Dehydrated lung tissues were sectioned into 4 μm thick sections after being embedded in paraffin (Shanghai Showbio Biotech, Inc., Shanghai, China). The sections were stained with hematoxylin and eosin (H & E) for light microscopic analysis using a Carl Zeiss Axio Zoom V16 microscope (Oberkochen, Germany). Two independent researchers evaluated each sample in a blinded manner. The extent of damage to alveolar walls was quantified using the DI, and the airspace size was measured using the MLI.

### 4.7. Cell Culture

Alveolar epithelial (A549) cells were cultured in 1640 Medium (Hyclone, Logan, UT, USA) supplemented with 10% fetal bovine serum (FBS, Invitrogen, Carlsbad, CA, USA), 100 U/mL penicillin, and 100 µg/mL streptomycin (Thermo Fisher Scientific, Waltham, MA, USA) at 37 °C in a 95% air and 5% CO_2_ atmosphere. Following the findings of a previous study, 10% CSE was applied to A549 cells at a density of 10^6^/mL per well for 12 h to induce cell damage. According to a prior study, A549 cells were exposed to 10% CSE for 12 h after cell attachment to mimic the lung cell destruction condition observed in COPD. To assess the effects of Luteolin on COPD, after successfully establishing the cell models, different groups were treated with various agents for an additional 12 h, and the groups were as follows: Control group (Control), treated with RPMI 1640 only; CSE group (CSE), treated with 10% CSE and RPMI 1640; Luteolin intervention groups, treated with 10% CSE, RPMI 1640, and different doses of Luteolin at 15 μM (Lut-L) and 30 μM (Lut-H). The Luteolin dosages in the cell experiment were based on our preliminary investigations.

### 4.8. Measurement of Ca^2+^ Influx

The Fluo-4 AM calcium assay kit (Beyotime, Nanjing, China) was employed following the manufacturer’s instructions to measure intracellular Ca^2+^ levels. Briefly, cells were seeded in LabTek 8-well chambers with glass bottoms (Fisher) at 1 × 10^6^/mL density. After three rounds of washing, cells were stained for 30 min at 37 °C in the dark with 5 μM Fluo-4 AM. The fluorescence intensity was subsequently assessed using a Carl Zeiss LSM 900 microscope after the cells had been rinsed three times with PBS. Carl Zeiss Zen software (Zen 2011, Carl Zeiss Microscopy, Germany) was utilized for image acquisition and quantification.

### 4.9. Measurement of Intracellular ROS and Mitochondrial ROS

The DCFH-DA (Beyotime, Nanjing, China), fluorescent plate reader, and fluorescence microscopy were used to measure intracellular ROS production. Cells were seeded on 96-well black plates with six parallel wells in each group at a 1 × 10^6^/mL density. A 10 μM DCFH-DA solution was used to stain cells for 15 min at 37 °C in the dark. Following three rounds of washing with serum-free DMEM, the level of ROS was assessed using a microscope (Carl Zeiss LSM 900). Mito SOX Red (Invitrogen), a redox-sensitive fluorescent probe specifically localized to the mitochondria, was employed to evaluate the abundance of mitochondrial ROS generation. A549 cells were treated with a 5 μM Mito SOX Red probe for 10 min at 37 °C. The red fluorescence was recorded using a microscope after the cells had undergone two PBS washes. Carl Zeiss Zen software was used for image acquisition and quantification. Red fluorescence was measured using a microscope after the cells had experienced two PBS washes, and Carl Zeiss Zen software was employed to acquire and quantify images.

### 4.10. Western Blotting

A test assessed the expression of TRPV1, SIRT6, CYP2A13, NRF2, PGC1, SOD1, and SOD2. To determine total proteins, a BCA protein assay kit was used to lyse homogenates of mice lung tissues or collected A549 cells for 30 min. The total protein samples were separated on a 10% SDS-PAGE, transferred to a PVDF membrane, and blocked with 5% non-fat milk in Tris-buffered saline with Tween 20 before being incubated against TRPV1, SIRT6, CYP2A13, NRF2, PGC1, SOD1, and SOD2 for an overnight incubation at 4 °C. The membranes were treated with secondary antibodies, HRP-conjugated goat anti-rabbit IgG (1:30,000, Cell Signaling Technology), for 2 h at room temperature. The Tanon 5200 Multi-imaging system (Tanon, Shanghai, China) was utilized to visualize a luminous signal after the membranes had been cleaned in TBST. Using ImageJ, the density of a particular band was measured, with GAPDH concentration serving as an internal control.

### 4.11. Statistical Analysis

Data were performed as mean ± SEM via GraphPad Prism (GraphPad Software Inc., La Jolla, CA, USA). One-way ANOVA with Tukey’s multiple comparison test was adopted to evaluate intergroup significance. *p* < 0.05 was considered statistically significant.

## 5. Conclusions

**Figure 12:**
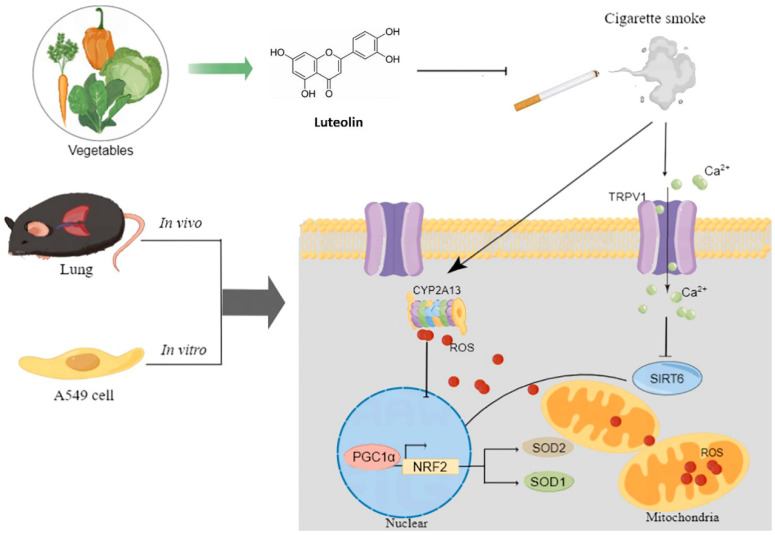
Schematic illustration of major points of conclusion. This graph was generated by Figdraw (www.figdraw.com, accessed on 1 September 2022).

In conclusion, both in vivo and in vitro studies provide significant evidence of Lut’s potential protective effects against CS-induced COPD. Lut increased body weight, restored lung damage, and attenuated systemic oxidative stress levels in CS + LPS-induced COPD mice. Additionally, Lut relieved oxidative stress and Ca^2+^ influx in CSE-exposed A549 cells. Ultimately, we demonstrated that the modulation of the TRPV1/SIRT6 and CYP2A13/NRF2 pathways may be the basis for Lut’s protective action against oxidative stress in CS-induced COPD (Figure 12). This study focuses on outlining the diverse protective mechanisms of Lut against COPD, specifically its capacity to alleviate oxidative stress and cellular damage in both in vivo and in vitro models. The study unveils a unique pathway by which Lut intervenes in the pathogenesis of COPD, presenting a fresh outlook on addressing this intricate condition. These discoveries propose a potential strategy for COPD treatment, broadening Lut’s medicinal applications. Nevertheless, our study has limitations. Subsequent research endeavors should involve utilizing mice of various genders, employing diverse cell lines, and assessing lung function to enable deeper exploration. Moreover, additional translational research is necessary to investigate and validate Lut’s therapeutic efficacy in clinical settings.

## Data Availability

The datasets used or analyzed during the current study are available from the corresponding author on reasonable request.

## Abbreviations

COPD	chronic obstructive pulmonary disease
CS	cigarette smoke
TRPV1	transient receptor potential vanilloid 1
ROS	reactive oxygen species
CYP2A13	Cytochrome P450 Family 2 Subfamily A Member 13
Lut	Luteolin
CSE	cigarette smoke exact
LPS	lipopolysaccharide
MDA	malondialdehyde
LDH	lactate dehydrogenase
SOD	superoxide dismutase
DCHF-DA	2′,7′-dichlorodihydrofluorescein diacetate
Mito-Sox	mitochondrial ROS
Ca^2+^	calcium ions
SIRT6	Sirtuin 6
NRF2	nuclear factor E2-related factor 2
PGC1α	peroxisome proliferator-activated receptor gamma coactivator-1α
MLI	mean linear intercept
DI	destructive index
CAT	catalase
GSH	glutathione
EGFR	epidermal growth factor receptor
MMP	matrix metalloproteinase
NOX4	NADPH oxidas
NAD^+^	nicotinamide adenine dinucleotide
NNK	nitrosamine ketone
H & E	hematoxylin and eosin
FBS	fetal bovine serum
DMSO	dimethyl sulfoxide
WB	Western blotting
ECL	enhanced chemiluminescence
